# Some Physiological Effects of Nanofertilizers on Wheat-Aphid Interactions

**DOI:** 10.3390/plants12142602

**Published:** 2023-07-10

**Authors:** Masoud Chamani, Bahram Naseri, Hooshang Rafiee-Dastjerdi, Javid Emaratpardaz, Asgar Ebadollahi, Franco Palla

**Affiliations:** 1 Department of Plant Protection, Faculty of Agriculture and Natural Resources, University of Mohaghegh Ardabili, Ardabil 5619911367, Iran; masoud.chamani@gmail.com (M.C.); hooshangrafiee@gmail.com (H.R.-D.); 2 Department of Agronomy and Plant Breeding, Faculty of Agriculture, University of Tabriz, Tabriz 5137779619, Iran; javid_emarat@yahoo.com; 3 Department of Plant Sciences, Moghan College of Agriculture and Natural Resources, University of Mohaghegh Ardabili, Ardabil 5697194781, Iran; ebadollahi@uma.ac.ir; 4 Department of Biological, Chemical and Pharmacological Sciences and Technology-Botany Section, The University of Palermo, 38-90123 Palermo, Italy

**Keywords:** nanotechnology, antioxidant enzyme, wheat, *Schizaphis graminum*, environmental health

## Abstract

The increasing use of nanofertilizers in modern agriculture and their impact on crop yield and pest management require further research. In this study, the effects of nano-Fe, -Zn, and -Cu (which are synthesized based on nanochelating technology), and urea (N) fertilizers on the antioxidant activities of wheat plants (cv. Chamran), and the wheat green aphid *Schizaphis graminum* (Rondani) are investigated. The authors observed the highest levels of phenolics in non-infested nano-Zn-treated plants (26% higher compared with control). The highest H_2_O_2_ levels are in the infested and non-infested nano-Zn-treated and infested nano-Fe-treated plants (in infested nano-Zn and nano-Fe treated plants, 18% and non-infested nano-Zn-treated plants, 28% higher compared with control). The highest peroxidase (POX) activity is observed in the infested and non-infested N-treated and non-infested water-treated plants (almost 14%, 37%, and 46% higher than control, respectively). The lowest activity is in the infested plants’ nano-Zn and -Fe treatments (almost 7 and 5 folds lower compared to the control, respectively). The highest and lowest catalase (CAT) activity are in the infested N-treated plants (almost 42% higher than control) and water-treated plants, respectively. The infested nano-Zn, -Fe, -Cu and Hoagland-treated plants showed the highest superoxide dismutase (SOD) activity. Regarding the antioxidant enzyme activities of *S. graminum*, the highest POX activity is in the nano-Cu treatment (more than two folds higher compared with control); the highest CAT and SOD activities are in the nano-Cu and -Zn treatments. It can be concluded that the application of nanofertilizers caused increasing effects on the wheat plant’s antioxidant system and its resistance to *S. graminum*.

## 1. Introduction

Wheat (*Triticum aestivum* L.) is one of the most important staple crops directly contributing to global food safety [1]. Numerous insect pests can damage this plant, leading to serious yield losses [2]. The wheat green aphid, *Schizaphis graminum* (Rondani), is one of the most devastating pests globally. The pest causes substantial economic damage by feeding the wheat plant and transmitting viral pathogens such as barley yellow dwarf virus (BYD) in most crops [3]. Its various biotypes can defeat resistant genes in wheat and sorghum and even detoxify pesticides [4]. Conventional pest control methods significantly contribute to applying insecticides to plants, resulting in significant risks to human and environmental security [5]. Thus, efficient alternative procedures, such as eco-friendly plant-derived agents [6,7], microencapsulation of pesticides, and nanotechnology, have been proposed to solve the issues mentioned above [2,8,9].

Recently, increasing demands for the application of nanotechnology in modern agriculture and environmental sciences have been witnessed, especially due to their exceptional and multipurpose characteristics [10,11]. Numerous studies have indicated the distinctive capability of nanomaterials concerning transportation through the cell wall and cellular membrane as functional barriers [12,13,14]. From this point of view, nanomaterials could be directly implicated in cellular biochemical pathways and reinforce them or act as carriers for other substances that may not be able to pass through the biological barriers alone [12,13].

Previous reports demonstrated the encouraging effects of nanomaterials on the efficiency of photosynthesis, the biosynthesis of secondary metabolites, the plant defense system, plant tolerance against environmental stresses, and the production of antioxidant compounds [15,16]. Since the absorption efficiency of nanomaterials is high, a smaller amount of the reagents would be needed. Accordingly, substituting traditional fertilizers with nanomaterials could decrease the probability of environmental pollution [17,18]. The localized application of high fertilizers such as ammonium salts, urea, nitrate, and phosphate compounds is detrimental. In any case, much of the fertilizers are unavailable to plants as they are lost as run-off leaching, which leads to pollution [19]. Thanks to the higher surface tension and slow release of nanofertilizers, plants could benefit more [20]. Moreover, nanofertilizers can either supply one or more nutrients to plants, resulting in enhanced growth and yield, or facilitate the better performance of conventional fertilizers without directly providing crops with nutrients [21]. Chelated fertilizers generally provide more benefits than other fertilizers, including high solubility, high stability, less environmental pollution, high efficiency in alkaline soils, high and fast mobility in plant roots and leaves, and rapid penetration into the plant [22].

Plants affect herbivores’ survival, nutrition, and reproduction through their biochemical, molecular, morphological, and physiological defense systems. Biotic and abiotic stresses cause opposing impacts on plant processes through changes in physiological and biochemical routes [23]. In this context, accumulating oxidative factors, mainly ROS (Reactive Oxygen Species) and free radicals, is nearly inevitable in all kinds of stresses.

The above-mentioned forms of reduced atmospheric oxygen with a high potential of reduction/oxidation (Redox) could induce lipid peroxidation, membrane damage, tissue necrosis, and other physiological disorders [24]. The increase in the activity of antioxidant enzymes in nanofertilizer-treated plants showed the importance of micronutrients in improving the growth and resistance of plants under stress conditions [25]. The most crucial antioxidant enzymes in both plants and animals are superoxide dismutase (SOD), peroxidase (POX), and catalase (CAT), which are activated during stress to eliminate ROS and H_2_O_2_ [26,27].

Plant phloem sap contains a ‘predigested’ food with a high concentration of sugars, providing an abundant source of carbon, nitrogen, and energy [28]. The growth and fecundity of herbivores, particularly sap-sucking insects, are generally influenced by plants’ nitrogen (N) content. Often, nitrogenous compounds are abundant in plant tissues, particularly in phloem sap [29].Therefore, aphids show a straight reaction to changes in the N level of host plants [30]. It has been demonstrated that applying nitrogen to wheat and oat plants can increase *S. graminum* population density [31]. Other nutritional materials have negative impacts on the reproduction and fertility of pests. For example, the fertility and fitness of the cabbage aphid (*Brevicoryne brassicae* L.) decreased by approximately 30% in plants treated with Cu and Pb [32]. Also, applying Zn led to detrimental effects on *Spodopteralitura* (F.) reproduction [33].

Advance chelate compounds technology (chelating and nanochelating) is a new method for producing compounds in different fields. In the last decade, studies proved the efficiency of nanofertilizers that were produced based on this technology. The scientific reports showed that using nano-chelated fertilizers can help plants to overcome against pests and stresses [34,35,36,37].

In the current research, we studied the effects of chelated nanofertilizers (Cu, Fe, and Zn) and N on the antioxidant system and biochemical properties of well-nourished wheat plants. We further attempted to elucidate the interaction of wheat-*S. graminum* under relatively high concentrations of nanofertilizer treatments by investigating the aphid antioxidant enzymes’ activity. Thus, the nature of the study was multidisciplinary. We developed new approaches to demonstrate how the physiological and biochemical properties of the host plant (wheat) could influence the enzymatic behavior of *S. graminum*. We also addressed the cascade of biochemical occurrences upon which the induced antioxidant enzymatic signal could be transported from the plant to this aphid. We aimed to investigate the effect of a relatively high concentration of mentioned nanofertilizers (Cu, Fe, and Zn) and N on metabolic processes. In addition, what is the effect of these substances on the antioxidant system of insects (part of the detoxification system), which can indicate the toxicity of these elements in insects, especially aphids?

## 2. Results

### 2.1. Total Phenolics Content

**Figure 1:**
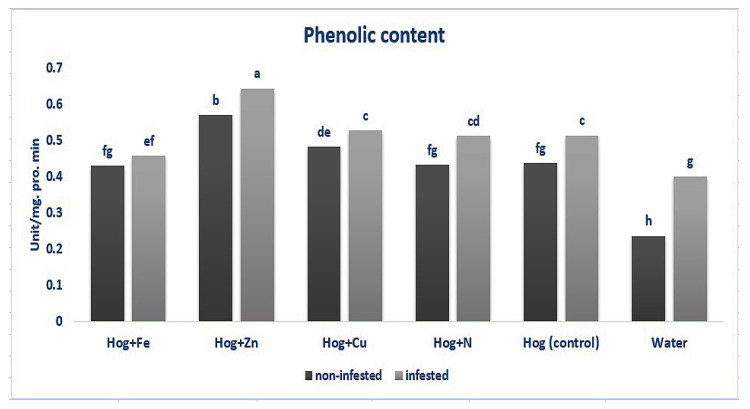
Phenolic content in wheat plants treated with Fe, Zn, Cu, N, Hoagland (control), and water under *Schizaphis graminum*-infested and non-infested conditions. Different letters demonstrated significant differences at *p* = 0.05.

Data analysis revealed that the plants treated with different fertilizers had enhanced phenolics content compared with the control (F_5,36_ = 26.881, *p* value < 0.01). Similarly, infesting the plants with the aphid led to an increase in the phenolics content compared with the control (except nano-Fe and water) (F_5,36_ = 26.881, *p* value < 0.01). Also, the application of nano-Zn caused a significant increase in the production of phenolic compounds in the infested plants compared with the non-infested ones (F_5,36_ = 26.881, *p* value < 0.01). No significant effects were observed regarding the N, nano-Cu treatments (F_5,36_ = 26.881, *p* value < 0.01). The highest level of phenolic compounds was exhibited in the plants treated with nano-Zn in infested and non-infested plants (F_5,36_ = 26.881, *p* value < 0.01) (Figure 1).

### 2.2. Wheat Lipid Peroxidation (MDA) Content

**Figure 2:**
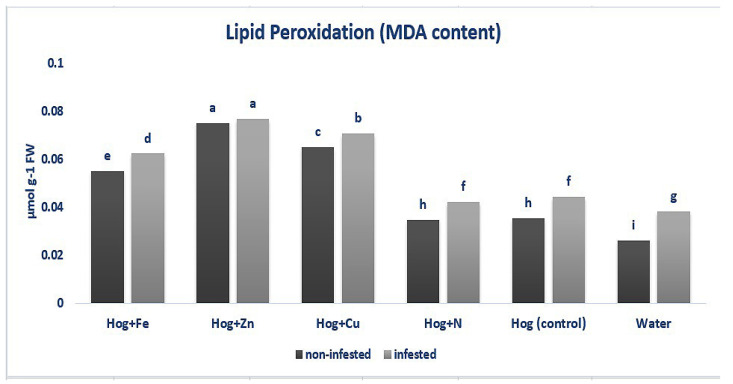
Rate of malondialdehyde (MDA) in wheat plants treated with Fe, Zn, Cu, N, Hoagland (control), and water under *Schizaphis graminum*-infested and non-infested conditions. Different letters demonstrated significant differences at *p* = 0.05.

The MDA content was enhanced in aphid-infested and non-infested plants (F_5,36_ = 7.906, *p* value < 0.01). In non-infested plants, treated plants with fertilizers exhibited significantly higher MDA content than the control (except N- and water-treated plants) (F_5,36_ = 7.906, *p* value < 0.01). Furthermore, without considering the nutritional status, the aphid-infested plants showed higher levels of MDA than the non-infested ones (except nano-Zn) (F_5,36_ = 7.906, *p* value < 0.01). The highest MDA level was observed in the nano-Zn-treated plants in both infested and non-infested plants (F_5,36_ = 7.906, *p* value < 0.01) (Figure 2).

### 2.3. Wheat Proline Content

**Figure 3:**
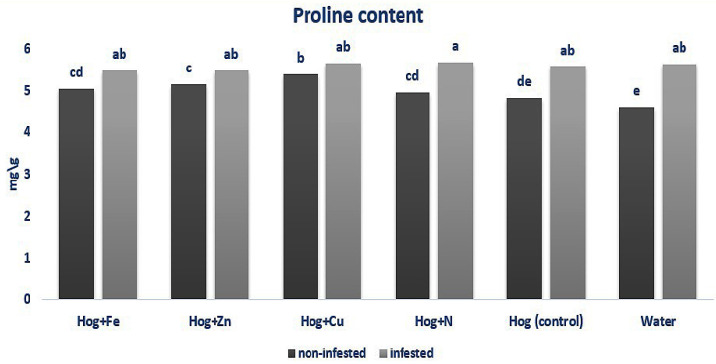
Amount of proline content in wheat plants treated with Fe, Zn, Cu, N, Hoagland (control), and water under *Schizaphis graminum*-infested and non-infested conditions. Different letters demonstrated significant differences at *p* = 0.05.

In non-infested plants, treating with nano-Cu and nano-Zn fertilizers caused a significant increase in the proline content compared with the control (F_5,36_ = 6.653, *p* value < 0.01). Also, plant infestation with aphids caused an enhancement in proline content compared with the non-infested ones (F_5,36_ = 6.653, *p* value < 0.01). Generally, aphid infestation has a higher impact on plant proline content than fertilization. However, significant differences were found in the aphid-infested and fertilizer-treated plants regarding proline amount (F_5,36_ = 6.653, *p* value < 0.01) (Figure 3).

### 2.4. Wheat Hydrogen Peroxide (H_2_O_2_)

**Figure 4:**
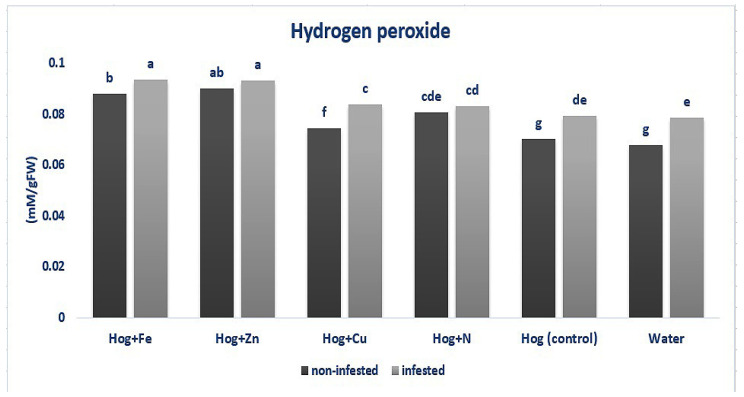
Amount of hydrogen peroxide in wheat plants treated with Fe, Zn, Cu, N, Hoagland (control), and water under *Schizaphis graminum*-infested and non-infested conditions. Different letters demonstrated significant differences at *p* = 0.05.

As shown in Figure 4, fertilizer treatments caused significantly higher H_2_O_2_ levels than the control, except for water treatment (F_5,36_ = 3.316, *p* value < 0.05). Also, H_2_O_2_ was enhanced in the aphid-infested plants compared with the control (except in N and water) (F_5,36_ = 3.316, *p* value < 0.05). Considering the N and nano-Zn treatments, no significant difference was recorded between the infested and non-infested plants (F_5,36_ = 3.316, *p* value < 0.05). On the other hand, the aphid-infested plants in Hog, nano-Cu, nano-Fe, and water treatments demonstrated higher H_2_O_2_ content than the non-infested ones (F_5,36_ = 3.316, *p* value < 0.05). The highest H_2_O_2_ content was obtained in the aphid-infested plants treated with nano-Fe and nano-Zn and in the non-infested nano-Zn treatment (F_5,36_ = 3.316, *p* value < 0.05) (Figure 4).

### 2.5. Antioxidant Enzymes Assay in Wheat

**Figure 5:**
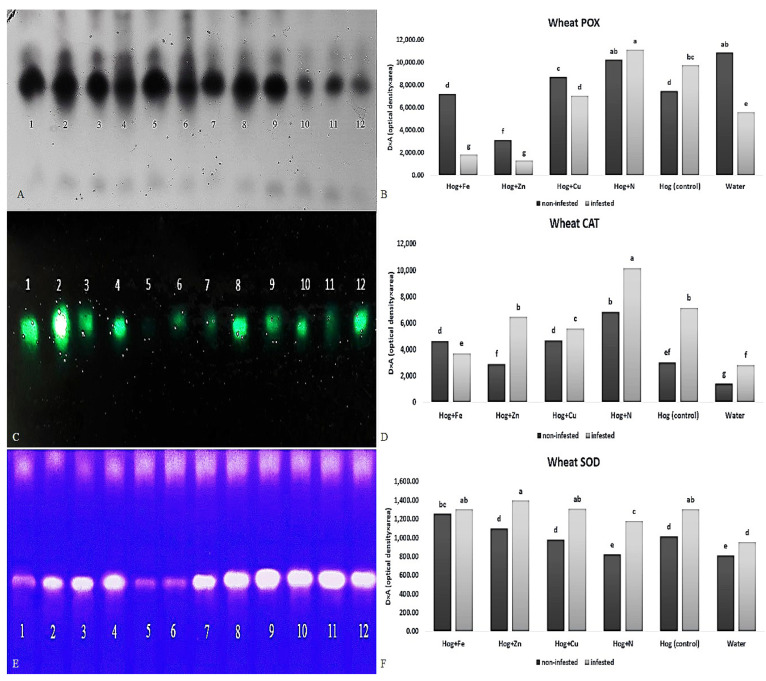
Native-PAGE (polyacrylamide gel electrophoresis) analysis of peroxidase (POX) (**A**,**B**), catalase (CAT) (**C**,**D**), and superoxide dismutase (SOD) (**E**,**F**) activities in wheat plants treated with Fe, Zn, Cu, N, Hoagland (control), and water under *Schizaphis graminum*-infested and non-infested conditions. Note: 1, 3, 5, 7, 9, 11 identifying N, nano-Cu, water, Hoagland (control), nano-Fe, nano-Zn treatments under non-infested condition, respectively; 2, 4, 6, 8, 10, 12 identifying N, nano-Cu, water, Hoagland, nano-Fe, nano-Zn treatments under aphid-infested, respectively. Different letters demonstrated significant differences at *p* = 0.05.

The infestation of wheat plants with aphids led to a decrease in POX and an increase in CAT and SOD enzyme activities (Figure 5). A similar trend in the changes in POX activity was not observed in treated plants under both infested and non-infested conditions. In non-infested plants, except for the nano-Fe and nano-Zn treatments, the application of the treatments enhanced POX activity (F_5,24_ = 61.757, *p* value < 0.01) compared with the control. The lowest rate was detected in nano-Zn-subjected plants, and the highest amounts were ascertained in the water and N treatments.

Infestation with aphids increased the rate of wheat POX activity in some fertilizer treatments such as N (F_5,24_ = 61.757, *p* value < 0.01). However, the application of nano-Fe and nano-Zn decreased the POX activity compared with the control (F_5,24_ = 61.757, *p* value < 0.01). The highest activity of POX was observed in the N treatment, and the lowest was seen in nano-Fe and nano-Zn (F_5,24_ = 61.757, *p* value < 0.01) (Figure 5A,B). Aphid infestation caused a significant decrease in wheat POX activity in some treatments compared with non-infested ones (F_5,24_ = 61.757, *p* value < 0.01). The POX activity in the water, nano-Fe, and nano-Zn treatments was significantly decreased.

All fertilizer treatments enhanced CAT activity (F_5,24_ = 52.694, *p* value < 0.01) in the infested and non-infested plants compared with the control (except in water and nano-Zn). The levels of CAT were higher in the aphid-infested plants than the non-infested ones under the fertilizer treatments, except for the nano-Fe treatment (F_5,24_ = 52.694, *p* value < 0.01). Compared with the non-infested plants, aphid infestation caused a significant increase in wheat CAT activity (F_5,24_ = 52.694, *p* value < 0.01) (Figure 5C,D). In control and nano-Zn treatments, the CAT activity was significantly increased (F_5,24_ = 52.694, *p* value < 0.01). The application of N caused the highest CAT activity in the infested plants (F_5,24_ = 52.694, *p* value < 0.01) (Figure 5C,D).

Applying all fertilizers had significant effects on the SOD activity (F_5,24_ = 19.703, *p* value < 0.01) in the non-infested plants. Additionally, aphid feeding caused an increase in SOD activity in all fertilizer treatments compared with non-infested ones (F_5,24_ = 19.703, *p* value < 0.01). Water-treated plants showed a lower activity of the SOD than the control (F_5,24_ = 19.703, *p* value < 0.01) (Figure 5E,F).

### 2.6. Antioxidant Enzymes Assay in Aphid

**Figure 6:**
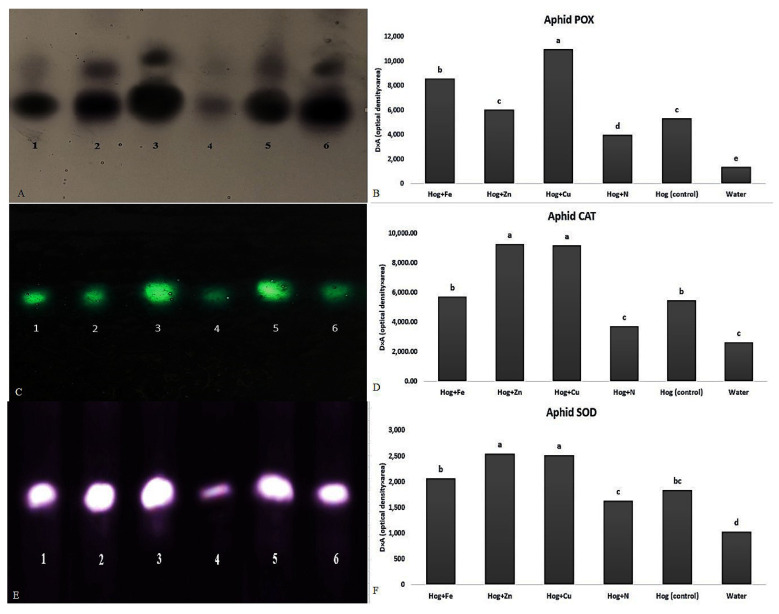
Native-PAGE (polyacrylamide gel electrophoresis) analysis of peroxidase (POX) (**A**,**B**), catalase (CAT) (**C**,**D**), and superoxide dismutase (SOD) (**E**,**F**) activities in *Schizaphis graminum* reared on wheat plants treated with Fe, Zn, Cu, N, Hoagland (control), and water. Note: 1, 2, 3, 4, 5, and 6 identifying the enzymatic activities of *S. graminum* reared on wheat plants treated with N, Hoagland (control), nano-Cu, water, nano-Zn, and nano-Fe, respectively. Different letters demonstrated significant differences at *p* = 0.05.

Almost all fertilizer treatments significantly increased the aphid POX, CAT, and SOD activities compared with the control (Figure 6). Concerning the applied treatments, the highest and lowest aphid POX activities (F_5,12_ = 30.878, *p* value < 0.01) were recorded on nano-Cu and N treatments, respectively (Figure 6A,B). Aphid POX activity in nano-Cu treatment was twice higher than in control (F_5,12_ = 30.878, *p* value < 0.01). Moreover, the highest CAT (F_5,12_ = 26.347, *p* value < 0.01) (Figure 6C,D) and SOD (F_5,12_ = 9.851, *p* value < 0.01) (Figure 6E,F) activities were observed in the aphids reared on the plants treated with nano-Cu and nano-Zn. The lowest activity was obtained in the aphids reared on the N- and water-treated plants. Aphid CAT activity on nano-Cu and nano-Zn was approximately 1.5-fold higher than its activity on the control (F_5,12_ = 26.347, *p* value < 0.01) (Figure 6C,D). Also, the CAT activity was almost 2.5 times higher than the activity on the N treatment. Aphid SOD activity on nano-Cu and nano-Zn was 2.5 times higher than that of the water (F_5,12_ = 9.851, *p* value < 0.01) (Figure 6E,F).

**Figure 7:**
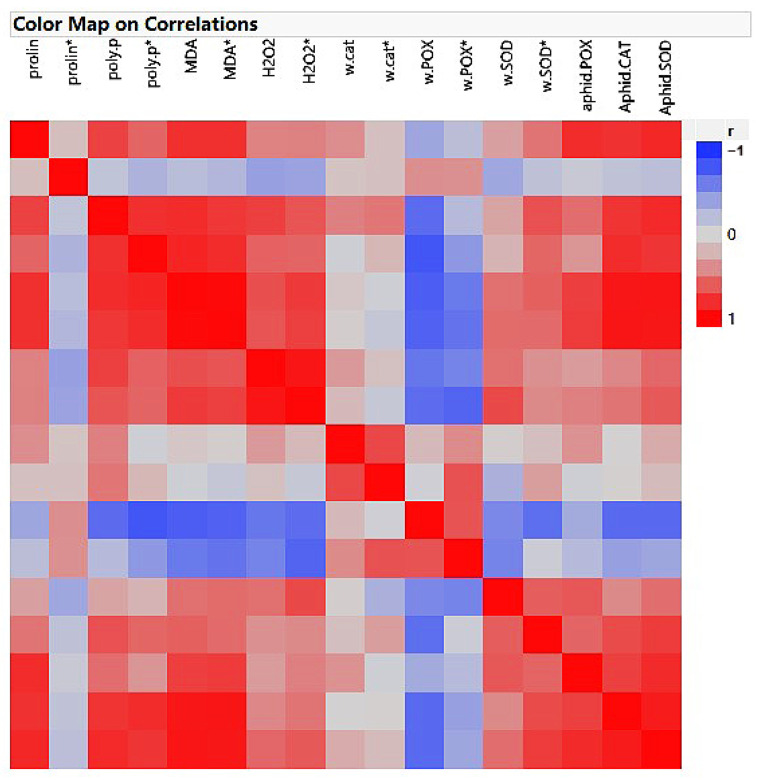
Heatmap of correlation in the non-infested, *Schizaphis graminum*-infested, and treated plants. Parameters marked with * were infested by the aphid.

As shown in Figure 7, the non-infested plants treated with all fertilizers demonstrated different correlations among the measured factors. The proline content showed a 70 and 79% correlation with phenolics and MDA, respectively. Phenolic contents had 80, 72, and 70% associations with MDA, H_2_O_2_, and POX, respectively. MDA displayed 64, 76, and 51% relationships with H_2_O_2_, POX, and SOD, respectively. Also, H_2_O_2_ demonstrated 63 and 51% correlations with POX and SOD, respectively.

Concerning the heatmap data in the infested and treated plants, phenolic compounds displayed 80, 57, 80, 55, 81, and 76% correlations with the MDA, H_2_O_2_, POX, SOD, aphid CAT, and SOD, respectively. MDA showed 71, 64, 53, 73, 92, and 90% correlations with H_2_O_2_, POX, SOD, aphid POX, CAT, and SOD, respectively. H_2_O_2_ had 73, 60, and 50% associations with wheat POX, SOD, and aphid CAT, respectively (Figure 7).

## 3. Discussion

**Figure 8:**
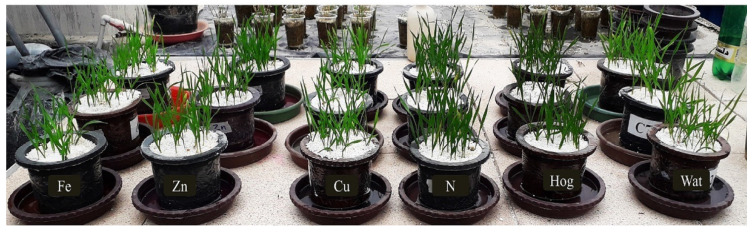
The appearance of plants treated with different nutrients (nano-Fe, nano-Zn, nano-Cu, N, Hoagland, and water).

According to the symptoms of the nanofertilizer-treated plants (Fe, Zn, and Cu) (Figure 8), the relatively high concentration of the elements probably caused abiotic stress compared to the control, which severely affected the plant antioxidant system and the secondary metabolites. In addition, water-treated plants experienced extreme stress due to nutrient deficiency. Furthermore, aphid activity on the treated plants altered the levels of secondary metabolites and enzymes that can indicate biotic and abiotic stress.

Adding micronutrients (i.e., Zn, Cu, and Fe) can induce oxidative stress, but plants have developed various defensive mechanisms, such as enzymatic (including CAT, POX, SOD, ascorbate peroxidase, and glutathione reductase) and non-enzymatic (including ascorbate, glutathione, flavonoids, phenolic compounds, tocopherol, and carotenoids) systems, that allow the scavenging of free radicals [38,39]. Due to the novelty of this field, a few studies have been conducted on this topic.

Our results demonstrated that when plants were fertilized with nano-Zn, nano-Cu, and nano-Fe, the amount of phenolics increased. Consequently, it seems that when plants are infested with aphids, phenolic compounds are available to eliminate radicals produced by aphid feeding. In other words, when biotic and abiotic stresses combined, phenolics increased to eliminate ROS. Phenolics are water-soluble antioxidants with the potential to protect plant cells, besides other critical roles [23]. They could also improve the efficiency of mineral absorption and accelerate the mobilization of elements in plants [40].

Furthermore, some studies have indicated that phenolic compounds substantially protect plants from oxidative stress under metal stresses [41,42,43]. So, it can be concluded that nanofertilizers, in a small amount, may derive plants by increasing phenolics and consequently increasing the absorption of elements. Additionally, increased phenolics may enhance plant resistance to pest attacks and high concentrations of elements. However, the increase in phenolics is not always beneficial to plants. For instance, accumulations of phenolics in plant tissues cause a reduction in plant growth and diminished carbohydrate and N content in phloem sap. However, this could be a restriction factor for aphid feeding on the plants, which is confirmed by the current study. The ZnO NPs application with NaCl in rapeseed (*Brassica napus* L.) caused the enhancement of phenolics in the seeds [44].

According to research done on *Imperta cylidrica*, the amount of phenolics in the shoots increased after 21 days of copper exposure [45]. Several studies have demonstrated that Fe deficiency increases the amount of phenolics in plants and their secretion from the roots because phenolics in the rhizosphere cause the chelation of insoluble Fe in the rhizosphere and transfer it to the plant. It can be concluded that the low amount of phenolics in nano-Fe treated plants is due to the sufficient availability of Fe to plant [46]. Chrzanowski & Leszczyński [47] stated that infestation of winter triticale seedlings with *Sitobion avenae* F. induced phenolics and subsequently induced resistance. Kariyat et al. [48] reported that the flavonoids (as derivatives of phenolic compounds) in wild sorghum caused significant mortality and reduced population growth in *Rhopalosiphum maidis* Fitch. In general, due to the high level of phenolics in the nano-Zn treatment, this element seems to induce more resistance to biotic and abiotic stress factors.

Aphids, as an external enemy, cause damage to the cell wall and increase the MDA content of the plant [49]. Also, the fact that lipid peroxidation is high in the aphid-infested wheat plants fertilized with different nutrient solutions would deduce that the vigorous plants have a favorable inner condition for encountering external enemies. MDA is the most important indicator for lipid peroxidation of cell membranes [49], affecting other cellular components by damaging proteins, nucleic acids, and polysaccharides [50]. Previous studies verified that the levels of H_2_O_2_ and MDA increased in plant cells due to ROS accumulation, the main factor in polyunsaturated lipid oxidation [51,52]. Our results agree with the notion that the levels of MDA often correlate with the extent of aphid activity. In other words, a high level of Zn in plant tissues could influence the production of ROS and MDA levels [53]. Zhang et al. [54] reported that plants fertilized with urea demonstrated a decrease in the MDA content of their leaves. It seems that N prevents the destruction of cell walls in plants or is one of the reasons that makes plants suitable for aphid feeding. Similarly, the current study observed the highest amount of MDA in the aphid-infested plants treated with nano-Zn due to Zn role in antioxidant enzyme activities and MDA production. It can be concluded that the more complete the plant nutrition, the lower the MDA level, and the less cell damage.

Proline is the only proteinogenic secondary amino acid excluding amino groups produced by plants to regulate the osmotic situation during stress conditions. This protects the structure of macromolecules and membranes during severe dehydration [55]. Based on our results, using the nanofertilizers and aphid infestation caused an increase in proline content, which refers to proline’s antioxidant activity and proline role in plant stresses. Proline contents correlate indirectly with water content, stomatal conductivity, photosynthesis, and antioxidant enzyme activity. They have been used as indices of plant stress [56,57]. Proline lowers cytoplasmic pH, maintains the proper ratio of NADP^+^/NADPH in metabolism, increases various enzymes activities [58], and, importantly, acts as a source of energy, carbon, and nitrogen for tissue recovery under stress conditions [59]. The aphid-infested plants with enhanced proline content may contain lower water content to decrease aphid feeding and help plants cope with stress. Proline enhancement in the infested plants would increase resistance to stress conditions through better photosynthesis. Also, proline enhancement in the infested and fertilizer-treated plants demonstrated higher antioxidant enzyme activities, which agrees with the above-mentioned studies. During the application of micronutrients (including Fe), proline enhancement has been previously confirmed in rice plants [60,61], probably due to a decrease in the activity of the electron transport system [62,63], which resulted in the accumulation of NADH and H^+^. The higher the metal ion concentration, the higher the protein content in *Spirulina* and *Anabaena* [64,65]. This is probably caused by increased proline with metal ion chelating ability.

H_2_O_2_ in low concentrations is detoxified by the plant antioxidant system and acts as a signal transduction molecule in plant defense systems against stresses. At higher levels, H_2_O_2_ destroys the cell wall and also has a high tendency to DNA, proteins, carbohydrates, and lipids [66,67]. Our results showed that fertilization and aphid infestation increased the amount of H_2_O_2_, perhaps because of cell wall destruction and fertilizer effects. One of the side effects of the fertilizers utilized in the current study is their participation in photosynthesis, which enhances H_2_O_2_ levels. As essential parts of proteins and enzymes, Fe, Cu, and Zn are positively involved in photosynthesis, which may indirectly increase the levels of H_2_O_2_ due to the running of photorespiration [68]. The mechanism by which H_2_O_2_ is produced is well understood. It is produced in peroxisomes, and RuBisCO is the key enzyme in this process [69,70]. The fulfilment of photosynthesis in healthy plants is obligatorily tied to oxygenation as a form of H_2_O_2_ [71]. Superoxide anion and H_2_O_2_ act as wound signaling factors generated in the damaged tissues under biotic stress conditions [72], confirming our results.

Nonetheless, the balance between the generation and removal of H_2_O_2_ would determine if the molecule would act as a signaling factor or a destructive element [73,74]. In this regard, low levels of H_2_O_2_ would participate in wound stress signaling. However, high reagent levels could turn on the antioxidant system in the plant.

The significant correlation obtained in the current study for the levels of H_2_O_2_, the application of the nanofertilizers, and the aphid activity on the plants agreed with the above-mentioned subjects. Fe could improve the photosynthetic efficiency of plants [75]. Moreover, it is an essential part of proteins and enzymes, resulting in an important function in plants’ physiological and biological processes [68]. Consequently, attributable to the encouraging effects of Zn and Fe on photosynthesis, respiration, cellular metabolism, and antioxidant enzymes, the increase in the level of H_2_O_2_ in nano-Zn and nano-Fe treatments might be explained.

H_2_O_2_ and ROS are detoxified by the plant antioxidant system and several enzymes, including SOD, POX, and CAT, to control ROS levels and protect cells under suboptimal conditions [26]. In addition, wounding due to aphid activity could activate antioxidant enzymes to eliminate ROS from the injured cells and lessen their effects [72]. Therefore, augmenting the antioxidant enzymatic activity and ascending the levels of antioxidants could be assessed as a primary symptom of stress in plants, which causes an obvious diminish in their growth and induces resistance to the stress factors. In addition, the existence of Zn is correlated with the activity of antioxidant enzymes, which consequently increases the levels of ROS and MDA [51,76]. Some reports have indicated that Zn could act as a cofactor for antioxidant enzymes at low levels. However, higher concentrations of Zn lead to toxic effects by which the activity of the related enzymes could be increased, in line with the current findings to some extent. Cu, a redox-active transition metal in Fenton chemistry, catalyzes hydroxyl radical production. Likewise, Zn, a redox-inactive metal, disables the cellular antioxidant pool, disrupting the metabolic balance and then enhancing a load of ROS [77,78].

Similarly, upper Cu or Fe amounts enhanced ROS generation via the Fenton reaction [79,80]. Furthermore, several studies have shown evidence of stress in plants exposed to ZnO NPs-treated soils [21,81,82]. According to our findings, fertilizing with nano-Zn, nano-Fe, and nano-Cu could benefit the plant, leading to the relative activity of the antioxidant system and making the plant potentially resistant to biotic and abiotic stresses.

Our study indicated that the water-treated wheat plants are demonstrating high activity of POX, which probably refers to the accumulation of H_2_O_2_ through photorespiration and the attempt of the plant to remove ROS by exploiting the POX enzyme. The decrease in the POX activity in the infested plants (with any treatments) may be due to diminishing photosynthesis and reducing photorespiration, which may lead to a decrease in the H_2_O_2_ levels. In this situation, the production of H_2_O_2_ would be low.

Concerning the water-treated wheat plants, the high activity of POX probably indicated nutritional stress in the plant. The decrease in the POX activity of the infested plants receiving any treatments may be due to the plant’s inability to maintain its defensive state and survive. Generally, higher plants induce POX in response to the absorption of microelements (e.g., Cu, Zn, Cd, and Pb) [83]. The lowest POX activity in the infested nano-Zn and nano-Fe treatments probably refers to their higher amounts of H_2_O_2_ and even MDA leading to problems in their decomposition by the enzyme. CAT likely played an essential role in neutralizing H_2_O_2_ in these treatments.

The CAT is the main antioxidant enzyme inhibiting H_2_O_2_, and its activity is enhanced in plants under stress conditions [84]. The increase in CAT activity is considered an indicator of higher ROS production. However, in some cases, a decrease in the activity of antioxidant enzymes has been reported under stress conditions [85]. In the current study, N increased CAT activity in the aphid-infested wheat plants, which may be related to its role in enhancing aphid feeding and the polemical role of N, in which it increased CAT activity in some plants while decreasing it in others. The role of N in the oxidative state of the plant is very controversial; for instance, in some cases, high N concentrations enhance the antioxidant defense in plants [54,86] and decrease lipid peroxidation (MDA) [54]. In plants such as beet [86], maize [54], and poplar [87], N content increases CAT activity. Rengel & Graham [88] showed that Zn fertilizer reduced the CAT activity of wheat plants. Zn and Fe are critical parts of many fundamental enzymes, such as CAT and SOD, and also take part in the synthesis of chlorophyll and indole-3-acetic acid (IAA) [89,90].

As our findings demonstrated, the aphid-infested plants treated with nano-Zn and nano-Cu revealed higher levels of SOD activity. This might be related to the cupro-zinc property of SOD. It could occur due to the plant’s ability to maintain the homeostatic state of Zn under oxidative stress conditions, as confirmed by several subsequent reports in line with the current outcomes. Studies have stated that SOD, the first line of defense to scavenge ROS [91], is a cupro-zinc protein. Thus it becomes essential to maintain Zn homeostasis in plant cells to protect them from oxidative stress [92]. SOD and CAT are the most important antioxidant enzymes that are functionally related because SOD converts superoxide free radical (O_2_) to H_2_O_2_, which is removed by CAT [93]. The infested wheat plants receiving different nano-nutrients demonstrated increased SOD and CAT activities, with the highest activities in nano-Zn, nano-Fe, and nano-Cu, introducing the enzymes as the first line of defense against oxidative stress [94]. Enhanced activity of SOD was reported in algal chloroplasts under Cu application [95,96]. In addition, SOD activity was enhanced in *Scenedesmus bijugatus* exposed to different Cu concentrations [97]. Since Zn is one of the metal cofactors of the antioxidative enzyme superoxide dismutase (SOD), its application in higher dosages could initiate oxidative stress [98].

Insects need low micronutrients, but higher concentrations of these elements are toxic and cause stress [91,92,93]. High concentrations of elements cause destructive effects on different processes in animals, including survival, growth, reproduction, metabolism, and the innate immune system [99,100]. Several studies confirmed the importance of ROS-dependent immunity in the immune system of invertebrates for survival [101]. The effects of nanomaterials on the immune defense mechanisms of insects have been poorly investigated. Furthermore, using antioxidant systems in response to ROS is one of the primary techniques for analyzing the toxicity of these compounds. Several studies confirmed this [102,103,104]. As an illustration, utilizing CuO NPs on *Blapus sulcata* demonstrated destructive effects on the DNA. Also, it disrupted the antioxidant system, including increased SOD activity, on the other hand decreased CAT and acetyl cholinesterase (AChE) activities [103]. In invertebrates, hemolymph enzymes are essential in humoral defense [105]. Therefore, innate immunity and antioxidant enzymes have been used as indicators for stress tolerance in invertebrates and to determine the level of environmental pollution [106]. It has been reported that the influence of nanoparticles on antioxidant enzyme activity varies based on numerous aspects, such as size, concentration, and the treated organism [107]. The activity of antioxidant enzymes may extremely change during the onset of high concentrations of elemental exposure but return to normal levels after a few days [108]. It seems that in the presence of non-toxic amounts of micro-fertilizers, the antioxidant system of insects continued its activity. The increase in the activity of antioxidant enzymes in aphids under different nanofertilizer treatments might confirm the transfer of these substances into the food chain. The insect midgut is the main site of food absorption, where the metals are mainly concentrated. The main site for micronutrient accumulation in insects is fat bodies involved in several homeostatic functions, such as regulating the synthesis and storage of nutrients or providing several metabolic pathways [109,110].

In this study, the highest activities of antioxidant enzymes in aphids were frequently observed in nano-Cu and nano-Zn treatments. The following studies support the findings of current research: Abd El-Wahab and Anwar [111] indicated that Cu and Zn nanoparticles increased SOD enzyme activity in *Spodoptera littoralis* Boisduval larvae. Investigations conducted on *Drosophila* sp. [112] and *Glyphodes pyloalis* Walker [113] also established the effects of metal nanoparticles on the antioxidant activity of these insects. In *S. littoralis*, ZnO NPs increased the activities of SOD and CAT [107]. Gomes et al. [114] similarly showed that the SOD and CAT activities increased after one week of exposure to CuO NPs in the digestive gland of *Monochamus galloprovincialis* Olivier. According to our results, the nano-Zn treatment causes an increase in enzyme activity, which confirms the critical role of Zn in enzyme function. A conclusion that may be drawn is that, in addition to the positive role of Zn in insects’ metabolism, the utilization of nano-Zn acts as an abiotic stress agent for insects and may be beneficial to pest management. In insects, Zn plays a vital role in the synthesis of lipids, proteins, carbohydrates and the duration of larval and pupal stages [115]. Also, Zn inhibits ROS production by competing with transition metals, especially Fe [116].

Insects could minimize ROS impacts through the sequestration of Fe in non-reactive forms due to the effect of free Fe (or Cu) in the accumulations of superoxide and H_2_O_2_. Although low-molecular-weight cellular components may chelate Fe ions, these Fe chelates can still participate in Fenton-type reactions. Based on our findings, considering the low levels of antioxidant enzymes in nano-Fe treatment, it may be that nano-Fe was not toxic enough to the aphids because specific proteins are responsible for transporting and storing Fe in its non-reactive form in the most efficient way [117] in which “transferrin” is the major one in insects as well as vertebrates [118]. Besides, “ferritin” facilitates Fe transport and storage in insects. Ferritin preserves Fe in a non-toxic form at relatively high concentrations so that it can be used for the biosynthesis of Fe-containing proteins [118]. The application of FeSO_4_ on *Schistocerca gregaria* generated oxidative stress leading to macromolecule damage and higher antioxidant enzyme activities (e.g., SOD, CAT), presenting a somewhat negative effect of Fe-containing fertilizers on insect physiology [119].

Regarding function, the xylem and phloem are tightly associated in plants. In short, stem bundles and minor veins without the cambium of cereal, xylem and phloem interact more closely and directly [120,121]. Various studies have demonstrated that high concentrations of elements and micronutrients are transported through the phloem [122,123]. Zn has high mobility in the phloem [122], but Fe is less mobile than Zn [124]. Despite the absence of toxins in the phloem, aphids feed on phloem sap. Because of the high concentration of micronutrients in the phloem, they transfer to the insect body and cause toxicity; hence, this may be the reason for the antioxidant system activation in these insects. As the toxicity of nanoparticles has not yet been clearly studied and is, of course, not entirely understood, it is clear that they cannot be employed carelessly. It is obvious that micronutrients affect crop nutrition positively and induce plant resistance to biotic and abiotic stresses. The application of nanotechnology in agriculture, particularly manufacturing nanofertilizers or nanopesticides, has also improved their performance and efficiency. Considering that few studies have been done on environmental pollution and transmission of elements to higher food chains and that there is little understanding of the quantity and mechanism of accumulation of nanomaterials, more studies are needed. Also, a comparison should be made between conventional fertilizers and nanofertilizers, as well as their transmission speed and volume.

## 4. Materials and Methods

The study was conducted at the research greenhouse and laboratory of Experimental Plant Physiology, Faculty of Agriculture, University of Tabriz (Tabriz, Iran).

### 4.1. Plant Material

The plants were obtained by sowing wheat seeds (cv. Chamran) in medium-sized (3 L) pots filled with perlite (10 plants per pot). The pots were then transferred to the greenhouse and kept at 22 ± 1 °C, 60 ± 5% RH and 16:8 (L:D) h photoperiod. These seeds were irrigated with tap water every other day until germination. Afterwards, germinated plants were irrigated with half-strength Hoagland’s solution [125] for the initial stages of germination for up to seven days. Then full-strength Hoagland’s solution was used (for two weeks). The pots were washed every five days to remove excess ions. For treatment application, the plant was treated with nanofertilizers every other day. Plant samples were taken for biochemical assays one week after the application of the treatments (Figure 8).

### 4.2. Nanomaterials Tested

The exploited nanomaterials, including nano-zinc (Zn), nano-copper (Cu), and nano-iron (Fe), were obtained from Khazra Company (Tehran, Iran) (Figure S1 and Table S1), and added to the nutrient solution. In addition, N was added in the form of urea (Merck Co., Darmstadt, Germany) into the nutrient solution. Based on the manufacturing company’s instructions, we used the average high and low values of the recommended concentrations for each pot (Table 1). Water-treated plants (as a treatment of nutrient deficiency stress to compare nutrient excess treatments) received only tap water. Hoagland-treated plants are considered as a control for this experiment.

### 4.3. Insect Culture

**Figure 9:**
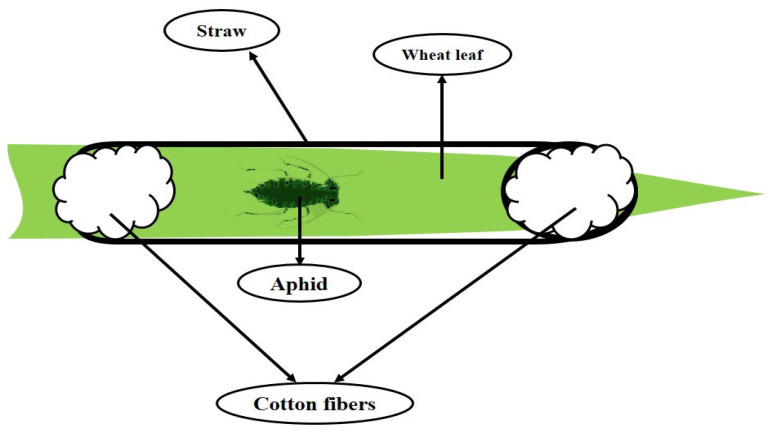
General scheme of limiting aphids on a wheat leaf.

The initial population of *S. graminum* was obtained from the previously existing colony in the Department of Plant Protection, University of Mohaghegh Ardabili, Iran, and reared on cultivated wheat plants (with three leaves). Infested plants were gradually replaced by healthy ones to ensure the availability of adequate aphids for the experiment. For the biochemical studies and investigating plant-aphid interactions, after one week of plant treatment, the leaves were infested with aphids, and feeding adult aphids on the leaves was enclosed using the plastic straw (0.5 × 10 cm) to avoid the aphids escaping from the target plants (Figure 9). All the assays were done with the same leaves and samples. The leaf samples were powdered using liquid nitrogen and then kept in a −80 °C freezer. Finally, all the assessments were done using these samples. All newborn nymphs were removed from the plants, and only primary mother entities were permitted to feed. Almost 72 h after feeding on treated wheat and control plants, 100 selected adult aphids were transferred into 1.5 mL microtubes and homogenized by a homogenizer. Then, the resulting mixture was centrifuged (15,000 rpm at 4 °C for 15 min), and obtained supernatant was used for the enzymatic assay by smearing Whatman^®^ papers and gelling. All measurements were performed in aphid-infested and non-infested conditions.

### 4.4. Total Phenolics Content

The total phenolics were measured (in four replicates) using the Folin-Ciocalteau reagent. Fresh, expanded leaves (0.1 g) were homogenized in 5 mL of 95% ethanol and centrifuged (10,000 rpm for 10 min). The digested mixture was kept in the dark, and then 1 mL supernatant, 1 mL of 95% ethanol, and 3 mL distilled water were mixed. Subsequently, Folin-Ciocalteau (1 mL) reagent, together with sodium carbonate 20% (5 mL) and deionized water (10 mL), was added to the prepared solution (100 mL). This mixture was completely stirred, and the absorbance was read at 750 nm using a spectrophotometer (UV-1800 Shimadzu, Kyoto, Japan). The phenolics content was expressed as mg per g of fresh leaves [126].

### 4.5. Lipid Peroxidation Content

Malondialdehyde (MDA) content, as an index of general lipid peroxidation, was measured (in four replicates) based on the colorimetric method [127,128]. Fresh, expanded leaf samples (0.1 g) were homogenized in 3 mL of trichloroacetic acid (TCA) 1% (*w*/*v*) at 4 °C. After centrifuging (15,000 rpm for 20 min), the same volume of TCA 20% was added to the test tubes at 96 °C for 30 min. Prepared extracts were then retained at 0 °C for 5 min, centrifuged (10,000 rpm, 5 min), and their absorbance was measured at 532 and 600 nm using a spectrophotometer. The MDA content was calculated based on the standardization procedure [128].

### 4.6. Proline Assay

The proline content (in four replicates) was assayed using standard phytochemical procedures with minor modifications [23]. For this purpose, samples from fully-expanded leaves (0.5 g) were homogenized in pre-cooled aqueous sulfosalicylic acid 3%. The provided solution was centrifuged (6000 rpm at 4 °C for 7 min), and supernatant (2 mL) was mixed with ninhydrin acid (2 mL) and glacial acetic acid (2 mL) and then incubated at 100 °C for 1 h. The reaction mixture was extracted with toluene under vigorous shaking for 20 s. The absorbance of the samples was determined at 520 nm by exploiting the spectrophotometer. The proline values were measured using the standard curve [129].

### 4.7. Hydrogen Peroxide Content

Fresh expanded leaf tissues (0.5 g) was extracted with 2 mL of 0.1% (*w*/*v*) TCA in an ice bath and subsequently centrifuged (12,000 rpm at 4 °C for 15 min). Afterwards, 0.5 mL potassium phosphate buffer (pH 7) and 1 mL potassium iodide (KI) were added to the 0.5 mL supernatant. The absorbance of the prepared solution was finally determined at 390 nm using a spectrophotometer, and the hydrogen peroxide content was measured based on the standard curve [130]. This measurement was done in four replications.

### 4.8. Native Polyacrylamide Gel Electrophoresis (Native PAGE)

The activity of antioxidative enzymes, including superoxide dismutase (SOD), peroxidase (POX), and catalase (CAT), was determined on a native PAGE. For this purpose, an extract of fresh leaf tissues was obtained using an extraction buffer (Tris-HCl, pH 7.5) based on the procedure described by Naderi, et al. [131]. Extracted samples were centrifuged (10,000 rpm at 4 °C for 10 min), at which point small pieces of filter paper (3 × 5 mm, Whatman^®^ 3) were immediately submerged. After absorbing the extract with papers, they were put in a 7.5% horizontal slab of the polyacrylamide gels (0.6 × 15 × 12 cm), which were prepared using Poulik buffer [132] and TBE (Tris-Borate-EDTA) electrode buffer (pH 8.8). Electrophoresis of the provided gels was carried out (4 °C, 3 h, 30 mA, 180 V), followed by staining of SOD and CAT [132], as well as POX [133]. Subsequently, the gels were fixed, and images were immediately acquired.

A similar method was fulfilled for the aphid samples. After the emergence of adult aphids on different treatments, 100 adults were randomly collected in 1.5 mL microtubes containing 0.5 mL distilled water. Then, the obtained samples were homogenized using a homogenizer and centrifuged (15,000 rpm at 4 °C for 15 min). The provided supernatant was used for the aphid enzymatic assay.

### 4.9. Statistical Analysis

The gel images were saved in TIFF format and analyzed using ImageJ software (ImageJ 1.52). The figures were drawn using Excel 2013, and the numerical data were imported into the statistical software SPSS ver. 20. The plant factors experiments were conducted on a factorial design based on a completely randomized design (CRD) at two levels (aphid-infested and non-infested). Analysis of variances was performed using two-way ANOVA and the means comparing was done using the Duncan Multiple Range Test (DMRT) at *p* = 0.05. The measurements of aphid antioxidant activity were carried out based on CRD, with DMRT to compare the means (at *p* = 0.05).

## 5. Conclusions

Considering the results of this study, nano-Zn application caused the highest antioxidant levels in wheat plants. In addition, the application of nano-Cu, nano-Fe, and nitrogen (N) activated antioxidant systems. All led to the hypothesis that the proportionate and combined use of nanofertilizers could enhance plant resistance to the pest; moreover, the toxicity impact of nano-Zn, nano-Fe, and nano-Cu on the aphid, identified by the activation of the insect’s antioxidant system, might demonstrate a reflection of the unfavorable influence of these elements on the aphid. More definitive results require more studies, particularly on the nanofertilizers effects on the life table, fertility, and length of the insect’s development, plus the environmental effects of the nanofertilizers application. However, it can be recommended that further research be carried out on the effects of relatively high element concentrations on wheat yield and the effects on aphid life table parameters, given the increasing effects that were found on the activity of antioxidant enzymes in aphids.

## Figures and Tables

**Table 1:** Fertilizer amounts recommended by the manufacturer per hectare and amounts used per pot in this experiment.

Fertilizer	Manufacturing Company Instruction (kg/ha)	The Amount Used per Pot (mg)
N (urea)	100	83.3
Zn (chelated zinc 12%)	4–6	6.25
Cu (chelated copper 8%)	1–2	1.87
Fe (chelated iron 9%)	3–10	8.12

## Data Availability

The data that support the findings of this study are available upon reasonable request.

## Supplementary Materials

The following supporting information can be downloaded at: https://www.mdpi.com/article/10.3390/plants12142602/s1, Table S1: Size of nano-chelated fertilizers used in the experiment; Figure S1: The transmission electron microscope (TEM) images of nano-chelated fertilizers (A) Fe nano-chelated fertilizer (B) Cu nano-chelated fertilizer (C) Zn nano-chelated fertilizer.
