# Supplementary material for: Some Physiological Effects of Nanofertilizers on Wheat-Aphid Interactions
Source: Plants (Basel). 2023 Jul 10;12(14):2602. doi: 10.3390/plants12142602 (PMC10385016; doi:10.3390/plants12142602)
Supplement: Supplementary file 1 [file plants-12-02602-s001.zip › plants-2450263-supplementary.pdf]

Supplementary Material

Table S1. Size of nano-chelated fertilizers used in the experiment

Nano-chelated fertilizer	Particle Maximum Size (nm)	Particle Minimum Size (nm)
Fe	20	30
Cu	45	60
Zn	35	45

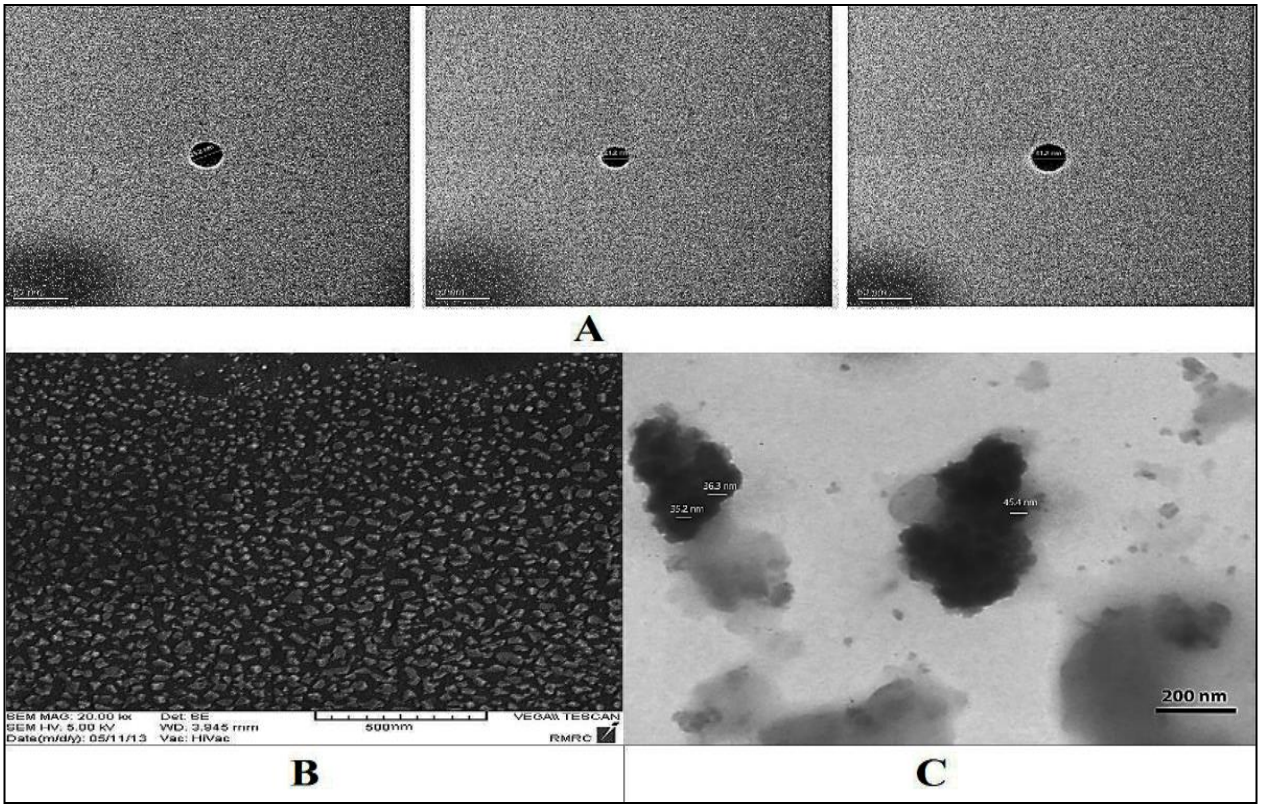

Figure S1. The transmission electron microscope (TEM) images of nano-chelated fertilizers (A) Fe nano-chelated fertilizer (B) Cu nano-chelated fertilizer (C) Zn nano-chelated fertilizer

Note: Nano fertilizers (Khazra) are produce based on nanochelating technology (Sodour Ahrar Shargh Co., Tehran, Iran). This technology is patented in United States Patent and Trademark Office (USPTO) and is based on "self-assembly" method.

The images of Fe nano-chelated fertilizer were captured by transmission electron microscope (TEM) at Melbourne University. According to these images, the particles size is approximately between 20-30 nm. In addition, based on TEM images which are captured in Tehran University, the particles size of Zn nano chelated fertilizer is 35-45 nm. The scanning electron microscope image at Tehran University showed that the porosity of the Cu nanochelated fertilizer particles is in nanometer dimensions and Cu fertilizer particles size is approximately between 45-60 nm.
